# Genitopatellar syndrome: the first reported case in Japan

**DOI:** 10.1038/s41439-018-0010-1

**Published:** 2018-05-28

**Authors:** Satomi Okano, Akie Miyamoto, Ikue Fukuda, Hajime Tanaka, Kenichiro Hata, Tadashi Kaname, Yoichi Matsubara, Yoshio Makita

**Affiliations:** 1 Department of Pediatrics, Asahikawa Medical University, Asahikawa, Japan; 2 Department of Pediatrics, Hokkaido Asahikawa Habilitation Center for Disabled Children, Asahikawa, Japan; 3 National Institute of Child Health and Development, Tokyo, Japan; 4 Education Center, Asahikawa Medical University, Asahikawa, Japan

## Abstract

Genitopatellar syndrome (GPS) is mainly characterized by an absence of patellae, congenital flexion contractures of the lower limbs, psychomotor retardation, and anomalies of the external genitalia and kidneys. We report an 18-year-old female with a novel heterozygous truncating mutation in exon 17 of the *KAT6B* gene [MC_000010.11:c.3603_3606 del, p.Arg1201fs]. This is the first report of typical GPS in a Japanese individual. The details of our findings may contribute to elucidating the mechanism underlying GPS-specific clinical features.

Genitopatellar syndrome (GPS, OMIM #606170) is a rare skeletal dysplasia manifested as genital hypoplasia, agenesis of the corpus callosum with microcephaly, and severe psychomotor retardation^1^. Since Cormier-Daire et al.^2^ first described the condition, less than 20 cases have been reported worldwide. A mutation in *KAT6B* (10q22.2), which encodes lysine acetyltransferase 6B, a part of the histone (H3) acetyltransferase complex, causes GPS^3^. Say-Barber-Biesecker-Young-Simpson syndrome (SBBYSS, OMIM # 603736), which is characterized by blepharophimosis, immobile mask-like face, lacrimal duct anomalies, and thyroid dysfunction, is an allelic disease also caused by *KAT6B* mutations^4^. Clinical features of both these diseases exhibit greater overlap (Table 1) than previously suggested; hence, a *KAT6B*-related disorder spectrum was considered^3^. Although SBBYSS mutations and overlapping features are located more broadly and distally in the large exon 18 of *KAT6B*^5^, mutations causing typical GPS cluster in the distal part of exon 17 to the proximal part of exon 18 between codons 1205 and 1350^6^. Herein, we present a typical case of GPS in a Japanese female with a mutation located near those reported in previous cases. To our knowledge, typical GPS has never been reported in Japanese individuals, with the exception of overlap syndrome^7^.

**Table 1:** Clinical manifestations of genitopatellar syndrome and Say-Barber-Biesecker-Young-Simpson syndrome

	**GPS**	**Common**	**SBBYSS**
Major features	※Genital anomalies ※Flexion contractures at hips and knees ※Agenesis of corpus callosum with microrocephaly ※Hydronephrosis or multiple renal cyst	※Patellar hypoplasia/agenesis	・Long thumb ・Immobile mash like face ・Lacrimal duct anomalies
Minor features	※Anal anomalies	※Congenital heart defect ※Global developmental delay ・Dental anomalies ・Hearing loss ・Thyroid anomalies ・Hypotonia	・Cleft palate ・Genital anomalies

※features　recognized　in　our　case

Campeau PM, Lee BH. *KAT6B*-Related

Disorders.GeneReviews^®^. Last Revision: January 10, 2013.

The patient is the first daughter of nonconsanguineous Japanese parents, with no family history of congenital anomalies. No abnormalities were identified by prenatal ultrasonography. She was born without asphyxia at 38 weeks and 1 day of gestation by caesarian section because of breech presentation. Her birth weight was 2775 g, her height was 44.3 cm, and her head circumference was 33.0 cm. These values were within normal limits. Peculiar face (Fig. 1a-c shows recent face images), wide thumbnails and wrinkled limbs (Fig. 1d), fracture of the right femur, dislocation of the left hip and both knees, and right clubfoot were recognized and required immobilization. X-ray imaging revealed bilateral missing patellae (Fig. 1e). Ultrasonography confirmed agenesis of the corpus callosum, dilation of the cerebral ventricle, atrial septal defect (7-mm diameter), mild mitral valve regurgitation, and peripheral pulmonary stenosis. She did not have an auditory disorder but required O_2_ during the ensuing months owing to laryngo-tracheomalacia. Her karyotype was 46,XX. During suckling, abdominal distention and vomiting occurred, requiring carminative treatment. Computed tomography revealed hiatal hernia, low anorectal anatomy, bilateral hydronephrosis, dysplastic kidneys (Fig. 1f), and lower sacral spina bifida. At age 5 months, gastrostomy and a radical operation for anal atresia were performed. During the operation, intestinal malrotation of the nonrotation type was identified, and the mesentery root was unroofed. The patient was discharged at 6 months of age. At 2 years, she underwent a tendon location operation and began taking medication for seizures. She suffered from ileus at 8 and 15 years of age.

**Fig. 1:**
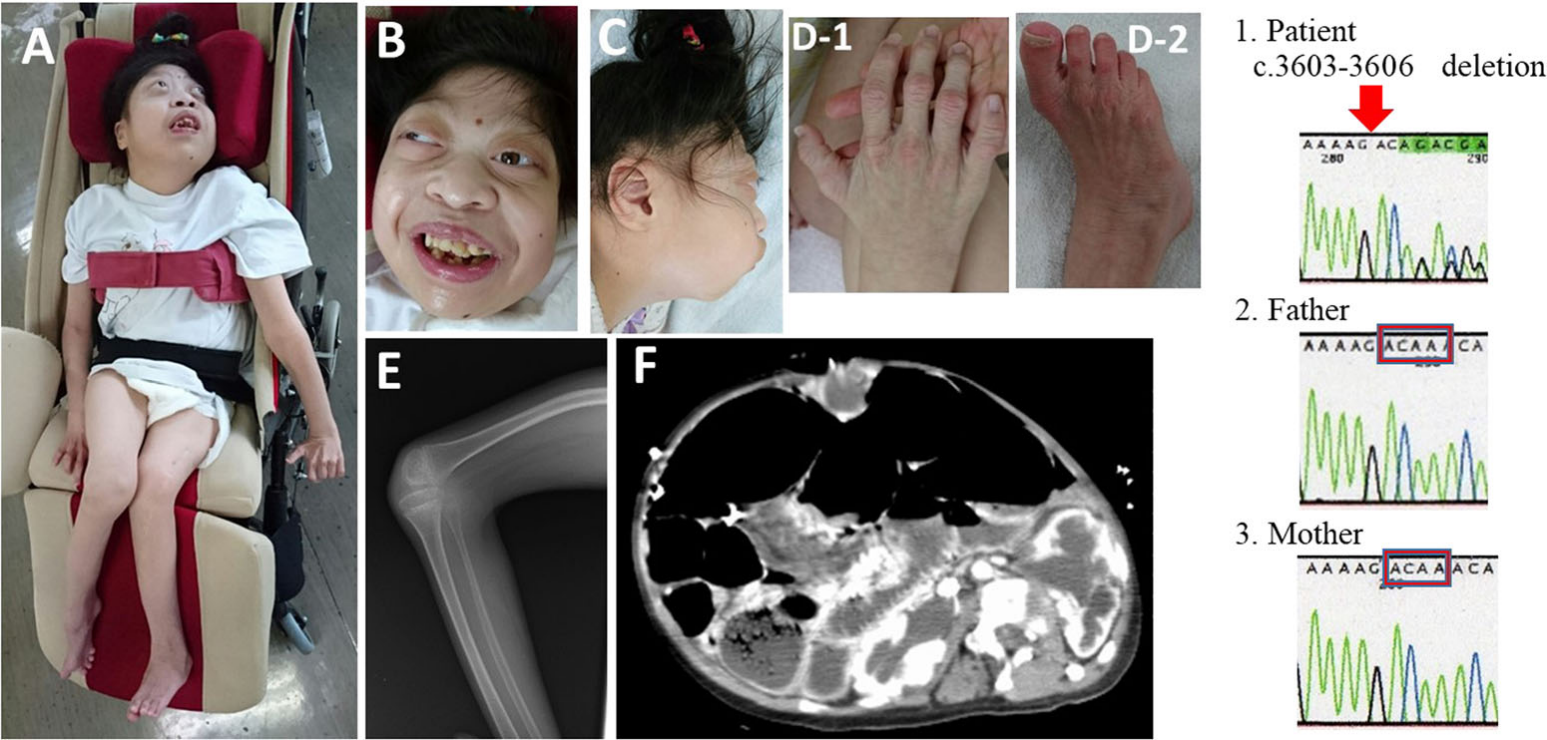
Current characteristic features of the patient and genetic analysis results. **a** Entire body of the patient. Developmental disturbance and right clubfoot are noted. **b** Coarse face with proptosis, right strabismus, and prominent broad nose. **c** Microcephaly and retromicrognathia. **d** Prominent middle and distal interphalangeal joints, wrinkled extremities, and broad thumb fingernails. **e** X-ray at 14 years of age reveals agenesis of patellae. **f** Contrast-enhanced computed tomography of the ileus at 15 years of age reveals pyelectasis and thin renal parenchyma. 1, 2, 3 Sanger sequencing reveals a c.3603-3606 deletion, a de novo, heterogeneous mutation in this patient

Currently, the patient is 18 years old and has severe psychomotor retardation without head control or verbal communication. She uses gastric gavage for nutrition. She presents with severe developmental disturbances: head circumstance 42.5 cm (equal to that at 7 months of age), height 121 cm (−7.8 SD, equal to that at 6 years of age), and body weight 24.0 kg (obesity index 5.0%). She has prominent middle and distal interphalangeal joints, single palmer creases, wrinkled extremities, and broad thumb fingernails (Fig. 1). Her genitals are abnormal, with hypoplastic labia majora, and she has not yet to undergo puberty or menarche. Blood tests revealed the following: luteinizing hormone 10.2 mIU/mL (follicular phase: 1.13–22 mIU/mL), follicle stimulating hormone 7.98 mIU/mL (follicular phase: 1.47–8.49 mIU/mL), estradiol 30 pg/mL (follicular phase: 22–147 mIU/mL), insulin-like growth factor 1134 ng/mL (18-year-old Japanese female normal range: 188–574 ng/mL), and prolactin 10.37 ng/mL (normal range: 4.91–29.32 ng/mL). Three years ago, her reproductive hormones were at prepubertal levels. Other blood findings were normal, including electrolytes, thyroidal function, adrenocorticotropic hormone, and hydrocortisone. However, her renal function has been gradually deteriorating, so we follow her up meticulously with monthly urine analysis. In addition, ^99m^Tc-MAG3 scintigraphy revealed predictive values for her right and left glomerular filtration rates as 5.6 and 33.6 mL/min, respectively.

Scientific research of this patient was approved by the Ethical Committee of the Hokkaido Asahikawa Habilitation Center for Disabled Children (permission number 29-7). After written informed consent was obtained from her parents, genetic analysis was performed by the Initiative on Rare and Undiagnosed Diseases (pediatrics), the nationwide consortium by the Japan Agency for Medical Research and Development. Using mutational screening by next-generation and direct Sanger sequencing, we identified a novel de novo heterozygous mutation: c.3603_3606 deletion (p.R1201fs) in exon 17 of *KAT6B* (Fig. 1,1). This mutation is currently not listed in public databases, such as Exome Variant Server (http://evs.gs.washington.edu/EVS/) and Human Genetic Variation Browser (http://www.genome.med.kyoto-u.ac.jp/SnpDB/).

The mutation in our case, which is located 4 codons upstream of a previously reported region^8^ at an Asp/Glu-rich acidic domain (Fig. 2), is hypothesized to be disease-causing. This result was confirmed in silico. The GPS alleles are predicted to cause truncation mutations of *KAT6B* at a location before the serine-rich and methionine-rich transcriptional activation domains, which regulate the acetylation of the histone tetrameric complex^1,3,5,8,9^. These domains interact directly with the runt domain transcription factor *Runx2*, which is responsible for cleidocranial dysplasia. However, the effect of this interaction is unclear, potentially explaining the unique features of GPS. Campeau et al.^1^ hypothesized that the gain-of-function *KAT6B* mutations, caused by an altered binding affinity or dysregulated interaction with partners of *KAT6B*, might cause the specific symptoms of GPS, whereas loss-of-function mutations are related to its common features.

**Fig. 2:**
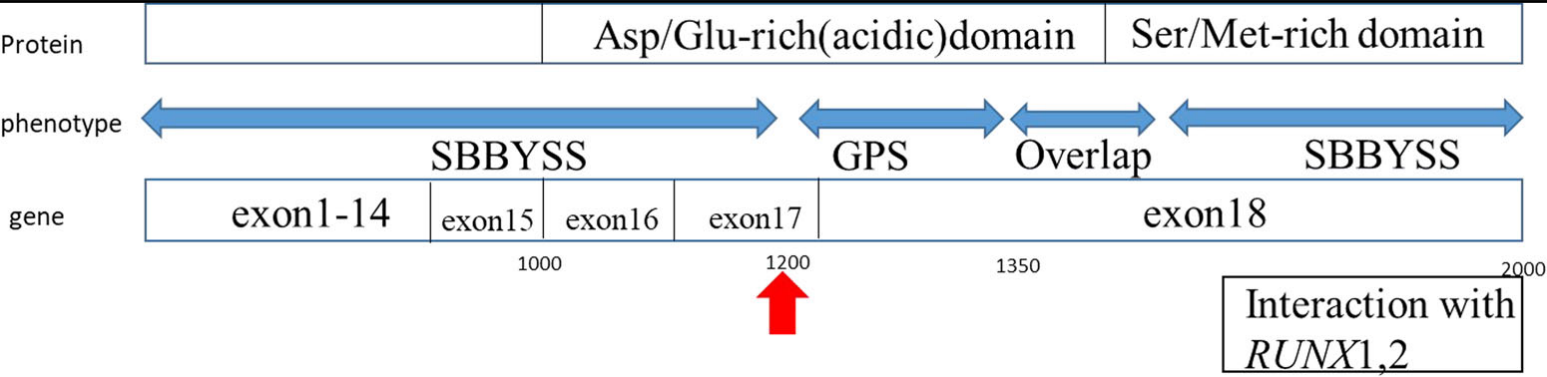
Schematic of KAT6B. Schematic of the *KAT6B* protein, gene and phenotype. A clumped distribution of typical genitopatellar syndrome at the Asp/Glu-rich acidic domain is obvious. The mutation in our patient (indicated by a red arrow) is located in a region neighboring the reported GPS region. The terminal region is mainly involved in the interaction of SBBYSS with *Runx*1 and *Runx*2

The oldest patient with GPS is a 25-year-old female without renal or cardiac anomalies^9^, making our patient the second oldest. Cormier-Daire et al.^2^ reported three patients who died during the first years of life owing to respiratory distress or sudden death. The long-term prognosis remains unclear and might depend on fetal complications of respiratory or congenital heart disease and kidney dysfunction. In our patient, the laryngomalacia was overcome, but renal impairment was gradually aggravated.

Penttinen et al.^10^ presented a 14-year-old girl without puberty. In our case, blood tests did not indicate hypergonadotrophic hypogonadism or pituitary-adrenal axis dysfunction. Although secondary sexual characteristics were not yet present, the secretion of reproductive hormones were constantly increasing, suggesting that the patient might be in a state of puberty onset.

In conclusion, we identified a novel truncating mutation of *KAT6B* in a female Japanese patient manifesting typical GPS features. The limitation of this report is that the cause-effect relationship is not well established. Further studies, such as functional analysis, may contribute to the identification of the mechanism underlying the distinct clinical manifestations and genotype-phenotype correlation of *KAT6B-*related disorders. Only 20 typical GPS cases have been reported to date; thus, the accumulation of more cases is expected to further our understanding of the mechanism.

## Data Availability

The relevant data from this Data Report are hosted at the Human Genome Variation Database at 10.6084/m9.figshare.hgv.1943.
